# Alendronate reduces periosteal microperfusion in vivo

**DOI:** 10.1016/j.heliyon.2023.e19468

**Published:** 2023-08-25

**Authors:** Danielle N. Kundert, Frank Tavassol, Andreas Kampmann, Nils-Claudius Gellrich, Daniel Lindhorst, Marc M. Precht, Paul Schumann

**Affiliations:** a Division of Cranio-Maxillo-Facial and Oral Surgery, University Hospital Zurich, University of Zurich, Frauenklinikstrasse 24, 8091, Zürich, Switzerland; b Department of Oral and Maxillofacial Surgery, Hannover Medical School, Carl-Neuberg-Strasse 1, 30625, Hannover, Germany; c Kieferchirurgie-Zentrum-Hamburg, Lerchenfeld 14, 22081, Hamburg, Germany

**Keywords:** Bisphosphonates, MRONJ, Microcirculation, Intravital fluorescence microscopy, Lewis rats

## Abstract

**Objectives:**

Bisphosphonates are known to induce a severe adverse effect known as medication-related osteonecrosis of the jaw (MRONJ). Previous studies have proven the impact of bisphosphonates on microperfusion; therefore, this study aimed to investigate alendronate-induced microcirculatory reactions in the calvarial periosteum of rats.

**Study design:**

Bone chambers were implanted into 48 Lewis rats. Microhemodynamics, inflammatory parameters, functional capillary density and defect healing were examined after alendronate treatment for two and six weeks using repetitive intravital fluorescence microscopy for two weeks.

**Results:**

Microhemodynamics remained unchanged. In alendronate-treated rats, inflammation was slightly increased, functional capillary density was significantly reduced (day 10: controls 100.45 ± 5.38 cm/cm^2^, two weeks alendronate treatment 44.77 ± 3.55 cm/cm^2^, six weeks alendronate treatment 27.54 ± 2.23 cm/cm^2^) and defect healing was decelerated. The changes in functional capillary density and defect healing were dose-dependent.

**Conclusion:**

The bisphosphonate alendronate has a significant negative impact on periosteal microperfusion in vivo. This could be a promising target for the treatment of MRONJ.

## 1 Introduction

Bisphosphonates are commonly used for the treatment of osteoporosis, bone metastases and multiple myeloma because of their antiresorptive effects that inhibit the activity of osteoclasts [[1], [2], [3]]. Despite good general tolerability they can provoke osteonecrosis of the jaws, first described in 2003 and called medication-related osteonecrosis of the jaw (MRONJ) [4,5]. The pathogenesis of MRONJ seems to be multicausal, depending on factors such as the type and dosage of bisphosphonates; predisposing factors such as diabetes, anaemia or corticosteroid therapy; and risk factors such as age or infection [6]. However, the precise molecular pathway of MRONJ pathophysiology remains unclear [7].

Bisphosphonates inhibit endothelial proliferation. Patients with MRONJ show a decrease in circulating endothelial progenitor cells and an increase in endothelial cell apoptosis due to the reduction of vascular endothelial growth factor by bisphosphonates [8]. Thus, the antiangiogenic properties of bisphosphonates may contribute to the development of MRONJ [9]. Moreover, treatment with bisphosphonates has been shown to cause sterile inflammatory reactions and enhance leukocyte-endothelial cell interactions [10,11]. These effects might be due to an increase in pro-inflammatory cytokines such as IL-1 and TNF-alpha [12]. All of these factors may cause secondary infections and healing disturbances.

The periosteum is a highly vascularised tissue covering the bone and critically affects bone regeneration after any kind of trauma or infection by contributing to the blood supply to the cortical bone. Periosteal activity is believed to be of crucial importance in bone physiology and pathophysiology, and therefore, in the healing process [13]. A basic requirement for the preservation of periosteal function is the presence of adequate blood flow in periosteal vessels, because normal healing seems to be critically linked to the reestablishment of periosteal microcirculation [14,15]. Bisphosphonates are known to affect periosteal perfusion by inducing leukocyte-endothelial cell interactions [15,16]. A previous study demonstrated an increased number of adherent and rolling leukocytes in the postcapillary venules of the mandibular periosteum of bisphosphonate-treated mice compared with that in non-treated animals [12]. However, limited data are available regarding alterations in capillary blood supply following bisphosphonate treatment. This blood supply can be measured by the functional capillary density which is defined as the total length of blood-cell-perfused microvessels in an observation area [13]. Interestingly, functional capillary density of the iris has been shown to be decreased by systemic inflammations [17].

Therefore, the aim of the present study was to investigate the in vivo effects of the bisphosphonate alendronate on microhemodynamics, inflammation and the healing process in the periosteum with special regard to functional capillary density in calvarial bone chambers of rats along with intravital fluorescence microscopy.

## 2 Materials and methods

### 2.1 Animals

The study was carried out with male Lewis rats, 8–14 weeks of age, weighing 300–330 g (Charles River WIGA, Sulzfeld, Germany) in Crossbit Hannover.

All procedures followed the prescriptions and guidelines of the National Institute of Health for the protection of laboratory animals (accepted protocol ref. 33 9-42502-04-08/1562).

The animals were kept in separate cages at 22 °C, 60% air humidity and a 12 h light-dark cycle. Animals were fed standard pellets (Altromin, Lage/Westfalen, Germany) and had free access to water. The health and weight of the animals were evaluated daily before every microscopic procedure.

### 2.2 Bone chamber implantation

The construction and implantation of the chamber have been previously described in detail [13,18].

The external diameter was 11.5 mm; and that of the observation window was 8 mm. The observation field was approximately 36 mm^2^. The observation window was fixed with a circlip that could be removed for examinations. The height of the frame was 1.4 mm [18]. Briefly, animals were anaesthetised using an intraperitoneal injection of ketamine (Ketavet®, 100 mg/kg body weight, Parke-Davis, Germany) and xylazine (Rompun®, 5 mg/kg body weight, Bayer HealthCare, Germany). Subsequently, the periosteum was exposed. The collagenous connective tissue was carefully excised using microsurgical instruments and a 3D microscope (Zeiss microscope, Zeiss Fluoartic, Germany) until the vascular layer of the periosteum was exposed. The frame of the chamber was placed on the periosteum and sutured to adjacent skin in such a way as to prevent drying (Ethicon Vicryl sutures 5–0, Johnson & Johnson, Germany). Finally, the cover glass was secured to the frame with a circlip [13].

### 2.3 Setting the periosteal lesion

To investigate the healing of the periosteal defects, periosteal lesions were set with an electrosurgical knife (MD62, Setting 5, KLS Martin, Tuttlingen, Germany). The lesion was standardised, being 1.5 mm wide and 6 mm long, using a positioning device. The device was a small titanium plate shaped identical to the observation window of the chamber and fitted precisely into it. In the centre of the device, a countersunk slot of 1.5 mm width and 6 mm length exposed the periosteum and enabled the setting of periosteal lesions of identical dimensions and positions in each animal of the group. This dimension was defined as the critical size in preliminary tests. After setting the lesion, the positioning device was removed and the operation field was rinsed with warm sodium chloride solution. The periosteum was not subjected to any further manipulation. During the period between examinations, the operation field was covered with the covering glass of the chamber and fixed using a circlip.

### 2.4 Intravital fluorescence microscopy

Intravital fluorescence microscopy was performed according to standard protocols [13,18].

Examinations were performed under anaesthesia with ketamine and xylazine immediately after implantation of the chamber and on days 3, 6, 10 and 14.

For high-resolution imaging of the microcirculation, fluorescein isothiocyanate-labelled dextran (FITC-labelled dextran, MG 150.000, Sigma Chemicals, USA) was injected for contrast enhancement by intravascular staining of blood plasma and rhodamine 6G (MG 476; Sigma Merck, Germany) was injected for direct in vivo staining of leukocytes. Immediately before each examination, 0.5 ml of FITC-labelled dextran (150 mg/ml 0.9% NaCl solution) and 0.5 ml of rhodamine 6G (1 mg/ml 0.9% NaCl solution) were injected intravenously. Animals were then immobilised on a special plexiglass table to position the chamber under the microscope and minimise head movements caused by respiration.

Fluorescence microscopy was performed under epi-illumination using a modified microscope (Zeiss Fluoartic, Germany). A blue filter block (450–490 nm) was used for the detection of FITC-labelled dextran and a green filter block (530–560 nm) was used to visualise leukocytes labelled in vivo with rhodamine 6G. Microscopic images were recorded using a charge-coupled device video camera (Pieper, FK-6990 IQ-S, Germany) and transferred to a DVD recorder (LQ-MS 800, Panasonic, Osaka, Japan).

### 2.5 Microcirculatory analysis

Quantitative offline analysis of the DVDs was performed using the computer-assisted image analysis system CapImage (Zeintl, Heidelberg, Germany) according to a standard protocol [18]. In each animal, microhemodynamics, leukocyte-endothelial cell interaction and macromolecular leakage were assessed at a magnification of 20× in four different microvascular regions of interest (ROIs). In each ROI, 1–3 venules (inner diameter: 20–30 μm) were selected for measurements. The diameters (d) were measured perpendicular to the vessel path. The centreline red blood cell velocity (v) was analyzed using the line-shift method. Leukocytes were classified according to their interaction with the vascular endothelium as adherent, rolling or free-flowing cells, as previously described [19]. Adherent leukocytes were defined in each vessel segment as cells that did not move or detach from the endothelial lining within a specified observation period of 20 s and were measured as the number of cells per square millimetre of endothelial surface, calculated from the diameter and length of the vessel segment studied, assuming a cylindrical vessel geometry [20]. Rolling leukocytes were defined as cells moving with a velocity less than two-fifths of the centreline velocity and were measured as the number of cells per minute, passing a reference point within the microvessel.

Macromolecular leakage served as an indicator of microvascular permeability and was assessed after an intravenous injection of the macromolecular fluorescent dye FITC-labelled dextran 150.000 by densitometrically determining the grey levels in the tissue directly adjacent to the venular vessel wall (E_1_) and in the marginal cell-free plasma layer within the vessel (E_2_). The extravasation (E) was then calculated as E = E_1_/E_2_.

Functional capillary density was assessed at 200x magnification. The cumulative length of microvessels per observation area, given in cm/cm^2^ was calculated by computer-assisted analysis using CapImage software [21].

### 2.6 In vivo animal experiments

Bone chambers were implanted into 48 Lewis rats. The animals were randomised into six groups of eight rats each. In groups 1, 2 and 3, the chambers were implanted without setting a periosteal lesion, whereas in groups 4, 5 and 6, electrosurgical lesions were applied. The animals in control groups 1 and 4 did not receive any preoperative alendronate dose. Animals in groups 2 and 5 were treated with alendronate (alendronate sodium trihydrate, Sigma Aldrich GmbH, Munich, Germany) preoperatively for two weeks, and animals in groups 3 and 6 were treated for six weeks. The dose was administered subcutaneously at 200 μg/kg body weight per day [22,23]. Microhemodynamics, inflammatory response and functional capillary density were determined in groups 1, 2 and 3 using fluorescence microscopy on the day of chamber implantation and on days 3, 6, 10 and 14 after implantation. The size of the periosteal lesions was examined in the groups 4, 5 and 6 using an identical time protocol. The rats were euthanised on day 14 after chamber implantation immediately after the last microscopic procedure under intracardial anaesthesia with a lethal dose of ketamine and xylazine.

### 2.7 Statistics

Results are expressed as the mean ± SEM. Differences between groups were evaluated using one-way analysis of variance (ANOVA). Differences within groups were analyzed using one-way repeated-measures ANOVA. The Holm-Sidak test was used to isolate specific differences, considering a p-value of <0.05.

## 3 Results

### 3.1 Microhemodynamics

Microhemodynamic parameters (venular diameters and centreline red blood cell velocities) were constant and comparable in all experimental groups at all points in time. No significant differences were observed between the groups throughout the experiment (Table 1).

**Table 1:** Diameters and red blood cell velocities of selected venules.

	Day 0	Day 3	Day 6	Day 10	Day 14
*Diameters (μm)*					
Control	26 ± 3	24 ± 2	26 ± 4	23 ± 1	23 ± 3
Alendronate 2 weeks	27 ± 4	26 ± 2	26 ± 3	28 ± 3	28 ± 4
Alendronate 6 weeks	21 ± 1	22 ± 2	24 ± 2	22 ± 4	22 ± 2
*Blood cell velocity (mm/s)*					
Control	0.82 ± 0.06	0.79 ± 0.03	0.85 ± 0.06	0.82 ± 0.05	0.79 ± 0.05
Alendronate 2 weeks	0.89 ± 0.09	0.91 ± 0.05	0.85 ± 0.07	0.87 ± 0.05	0.92 ± 0.06
Alendronate 6 weeks	0.81 ± 0.07	0.89 ± 0.08	0.88 ± 0.06	0.84 ± 0.03	0.80 ± 0.06

Diameters and red blood cell velocities of selected venules in controls and rats treated with Alendronate for 2 weeks and 6 weeks immediately (day 0) as well as 3, 6, 10 and 14 days after calvaria bone chamber implantation. Means ± SEM.

### 3.2 Inflammatory parameters

**Fig. 1:**
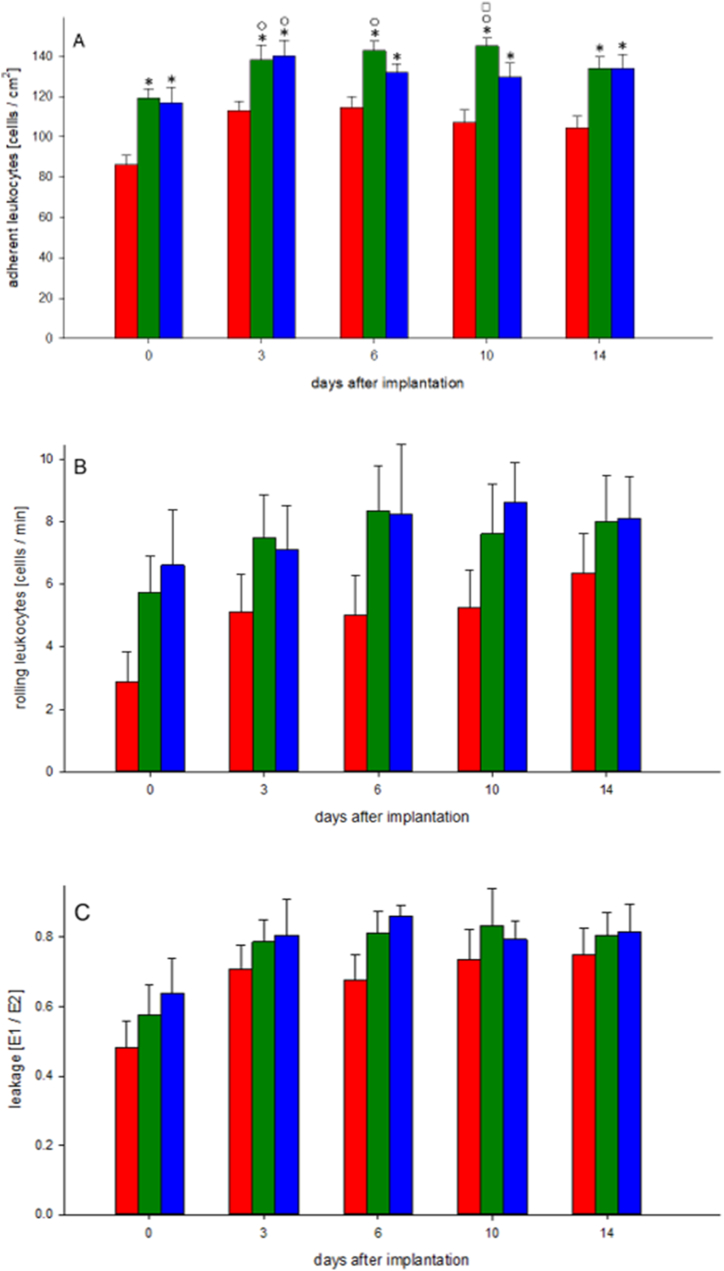
Inflammatory response of the periosteal tissue. The number of adherent leukocytes, given as cells/mm^2^ (A); the number of rolling leukocytes, given as cells/min (B); and macromolecular leakage as an indicator of microvascular permeability (C), immediately (day 0) as well as 3, 6, 10 and 14 days after implantation of the bone chamber (any group, n = 8). Data are presented as mean ± SEM. Control group - red bar; 2 weeks Alendronate treatment - green bar; 6 weeks Alendronate treatment - blue bar. A: *p < 0.05 versus control group on day 0. ⚬ p < 0.05 versus control group on day 10 and 14. ☐ p < 0.05 versus control group on days 3 and 6. B: The differences in the mean values among the treatment groups are not great enough to exclude the possibility that they are due to random sampling variability. So there is no statistically significant difference. C: No statistically significant differences.

**Fig. 2:**
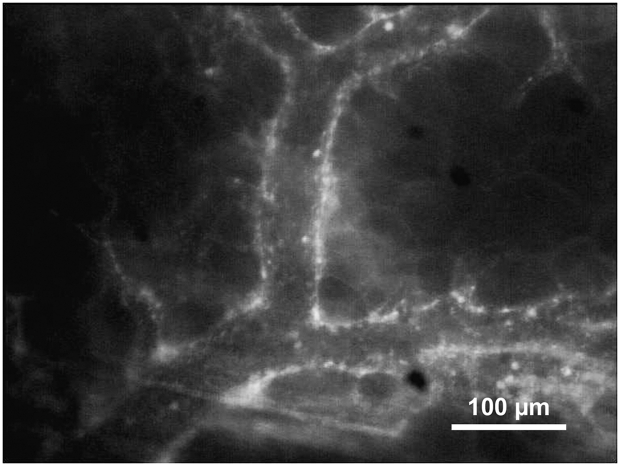
Intravital fluorescence microscopy of a postcapillary venule in a rat treated with Alendronate for two weeks 3 days after chamber implantation. Recruitment of leukocytes (bright spots) within the vessel lumen is demonstrated using green epi-illumination and rhodamine 6G for in vivo staining of white blood cells.

All inflammatory parameters (adherent and rolling leukocytes and macromolecular leakage) showed a slight increase between days 0 and 3; however, they remained at a nearly constant level during the rest of the examination period. Within each group, no significant changes were detected throughout the course of the experiment [Fig. 1(A–C) and 2] (see Fig. 2).

Animals treated with alendronate tended to have a more intense inflammatory response than the control group. Regarding adherent leukocytes, some of these differences were statistically significant [Fig. 1(A–C) and 2].

### 3.3 Functional capillary density

**Fig. 3:**
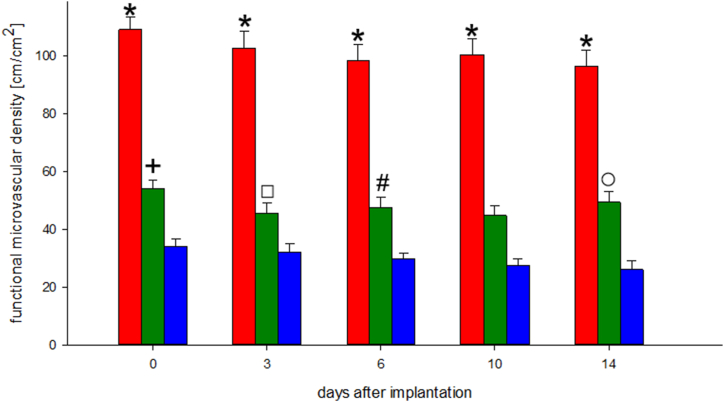
Time course of functional capillary densities, perfused capillaries. Quantitative assessment of functional capillary densities, given in cm/cm^2^, immediately (day 0) as well as 3, 6, 10 and 14 days after implantation of the bone chamber (any group, n = 8). Data are presented as mean ± SEM. Control group - red bar; 2 weeks Alendronate treatment (A2w) - green bar; 6 weeks Alendronate treatment (A6w) - blue bar. *p < 0.05 versus A2w and A6w on all examination days. + p < 0.05 versus A6w on all examination days. ⚬ p < 0.05 versus A6w on days 6, 10 and 14. #p < 0.05 versus A6w on days 10 and 14. ☐ p < 0.05 versus A6w on day 14.

**Fig. 4:**
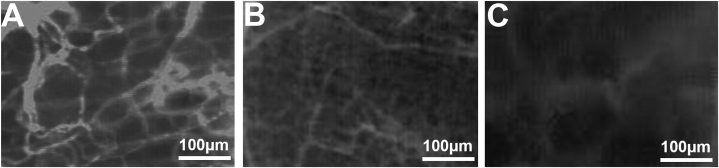
Intravital fluorescence microscopy of microvascular networks 6 days after chamber implantation (control - A, 2 weeks Alendronate treatment - B, 6 weeks Alendronate treatment - C). Blue light epi-illumination with contrast enhancement by addition of 5% FITC-labelled dextran 150.000.

The functional capillary density did not differ significantly within each group over the entire examination period. However, compared to the control group, functional capillary density was significantly reduced in the alendronate-treated groups. Compared with the control group, the total length of perfused capillaries reduced to half in animals with two weeks of therapy, and reduced to a third of the value after six weeks of therapy. Comparing the bisphosphonate-treated groups (2 weeks versus 6 weeks), there was an obvious trend towards less perfused capillaries after six weeks of therapy, showing significant differences in some cases [Fig. 3, Fig. 4 C].

### 3.4 Defect healing

The size of the periosteal lesion diminished continuously in all groups at all time points. However, the extent of defect healing was significantly reduced in the alendronate groups compared with that in the control group on all days of examination.

**Fig. 5:**
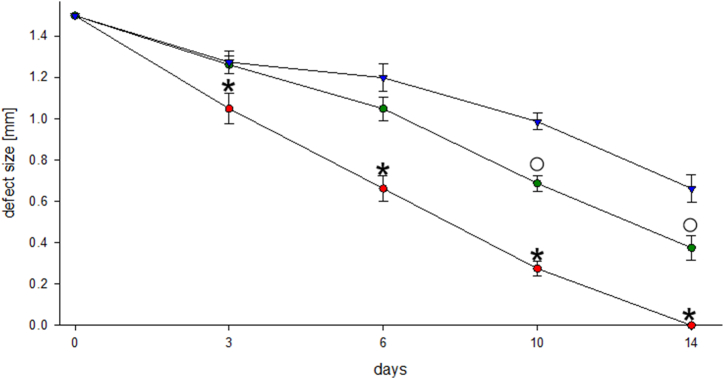
Time course of healing processes of the periosteal lesions. Quantitative assessment of defect size, given in mm, immediately (day 0) as well as 3, 6, 10, and 14 days after implantation of the bone chamber (any group, n = 8). Data are presented as mean ± SEM. Control group - red dots; 2 weeks Alendronate treatment (A2w) - green dots; 6 weeks Alendronate treatment (A6w) - blue triangles. *p < 0.05 versus A2w and A6w on the same day of examination. ⚬ p < 0.05 versus A6w on the same day of examination.

**Fig. 6:**
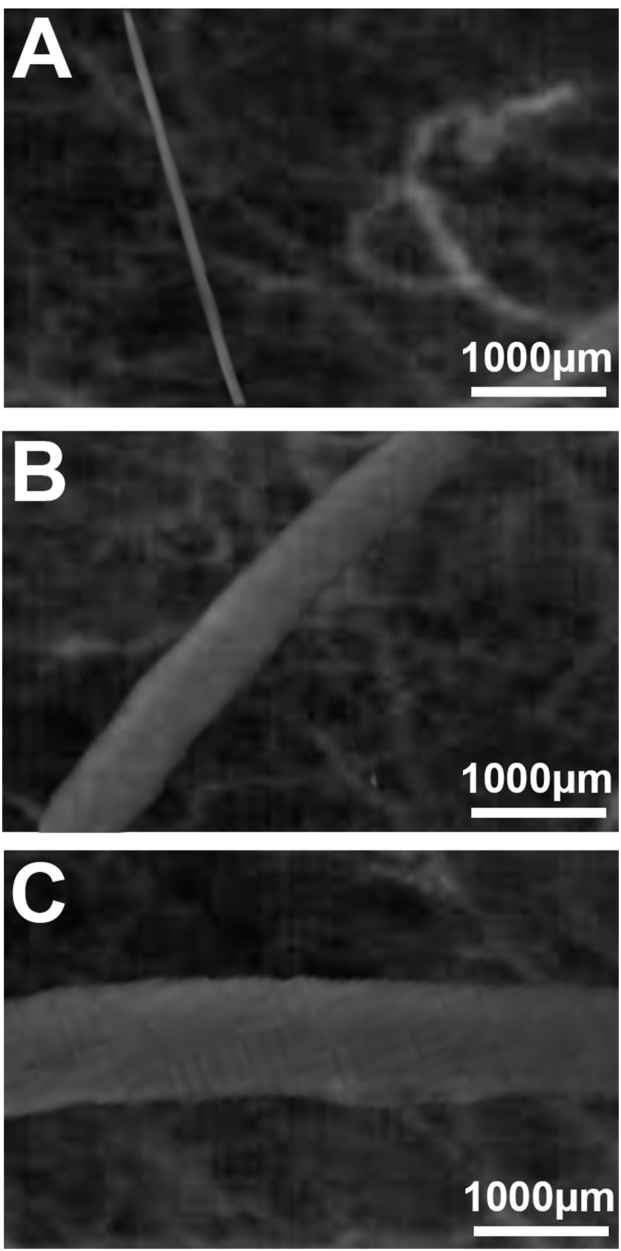
Intravital fluorescence microscopy of periosteal defects 10 days after chamber implantation (control - A, 2 weeks Alendronate treatment - B, 6 weeks Alendronate treatment - C). Blue light epi-illumination with contrast enhancement by addition of 5% FITC-labelled dextran 150.000.

In the control group, the width of the lesion was reduced to zero on day 14, whereas the size of the defect was still 0.38 mm in the two weeks treatment group and 0.66 mm in the six weeks treatment group. Within the alendronate-treated groups, defect healing was clearly reduced after six weeks of treatment compared with that after two weeks of treatment, showing a statistical significance on days 10 and 14 [Fig. 5, Fig. 6].

## 4 Discussion

In the present study, we demonstrated in vivo that treatment with the bisphosphonate alendronate increased the inflammatory response and reduced the functional capillary density and healing process in the calvarial periosteum of rats.

The method used to examine in vivo microperfusion in the calvarial periosteum of rats has already been described in detail [18]. It allows repetitive and quantitative analysis over an extended period (at least 14 days). The protocol of intravital fluorescence microscopy combined with bone or skinfold chambers has been shown in many studies to be a reliable method to examine in vivo microhemodynamics, inflammatory response and functional capillary density [20,[24], [25], [26], [27], [28], [29]]. Many studies so far have proven that periosteal microhemodynamics and perfusion are not influenced by the bone chamber or its implantation itself [13,18,30]. Consistently, the present study showed no remarkable changes in microvessel diameters and centreline red blood cell velocities between the control and alendronate-treated groups during the whole examination period. The vessel diameters and red blood cell velocities were comparable to those measured in previous studies [18,30]. Similarly, Janovszky et al. did not find any differences in the red blood cell velocity of periosteal venules in animals treated with zoledronate compared to controls [16].

We detected a more intense inflammatory response in animals treated with alendronate. An increase in leukocyte-endothelial cell interactions was observed in all groups between days 0 and 3. Alendronate-treated groups showed higher values, showing some significant differences in adherent leukocytes compared to controls. The slightly increased inflammatory response in all groups (elevation of rolling leukocytes, adherent leukocytes and macromolecular leakage) between days 0 and 3 may have been provoked by the surgical trauma of setting and manipulating the bone chamber. A comparable result was found in another study examining the periosteal vascularisation in the calvaria of rats using an identical bone chamber without bisphosphonate treatment but after bone augmentation [30]. Zysk et al. observed an increase in rolling and extravasated leukocytes in synovial microvessels of the knee joint in mice treated with ibandronate [12]. Janovszky et al. examined zoledronate-induced periosteal inflammatory reactions in the mandibles of rats after molar tooth extraction and found mandibular osteonecrosis in 70% of animals and healing disturbances in all zoledronate-treated animals. Triggered by the extraction trauma, zoledronate induces significantly higher degrees of rolling and adherent leukocytes in postcapillary periosteal venules [16].

Several studies have reported inflammatory reactions to bisphosphonates in the orbita [17,31,32] and bisphosphonate-induced irritation of the gastrointestinal tract [[33], [34], [35]]. The severity of the inflammatory side effects of bisphosphonates seems to depend on their chemical structure and clinical potency [36,37]. Keren et al. investigated the orbital/visual side effects of bisphosphonate therapy with zoledronate, pamidronate and alendronate. Compared with alendronate, zoledronate and pamidronate initiated more inflammatory side effects such as orbital diseases (periocular oedema, conjunctival hyperaemia and chemosis) and uveitis [38]. Shima et al. stated that nitrogen-containing bisphosphonates have more therapeutic potency than non-nitrogen-containing bisphosphonates, but cause more severe side effects such as inflammatory reactions and injury to oesophageal and gastrointestinal epithelial tissues. They inhibit the activity of farnesyl-pyrophosphate-synthase (FPPS) which leads to accelerated apoptosis of osteoclasts and other cell types [39,40]. Referring to this alendronate has a lower inhibitory effect on FPPS than that of zoledronate and pamidronate [41]. Nitrogen-containing bisphosphonates also enhance the induction of histidine decarboxylase (HDC), influencing the metabolism of histamine and, thus, the stimulation of proinflammatory cytokines [[41], [42], [43]]. Endo et al. ranked the potency of different bisphosphonates and stated that alendronate has a high affinity for hydroxyapatite, but a low inhibitory effect on FPPS [41]. Therefore, alendronate has a lower potency for inflammatory and necrotic effects than that of zoledronate and pamidronate [41]. Nevertheless, we detected a moderately increased inflammatory response in the alendronate-treated groups compared to that in the controls.

Concerning macromolecular leakage, it has been found that bisphosphonates inhibit the fibroblast growth factor (FGF) which is responsible for endothelial homeostasis and vascular integrity [44]. The lack of FGF seems to increase vascular leakage [45]. In addition to the slightly increased leukocyte-endothelial cell interactions, this could explain the elevated macromolecular leakage observed in our study.

Regarding vascularisation, alendronate significantly reduced periosteal functional capillary density in vivo. Zoledronate and pamidronate have been found to reduce perfusion in several experimental studies on rat limbs [46,47]. However, Wehrhan et al. showed using an immunohistochemical approach that in zoledronate-treated tissues, only neoangiogenesis was impaired, whereas local vascularisation was not significantly reduced [48]. Unaffected vascularisation in bisphosphonate-treated oral tissues was also reported by Hellstein et al. [49]. Microvascular density has been examined in the tibial, mandibular and calvarial periosteum of rats [50]. In untreated animals, the mean value of functional capillary density was 180 cm/cm^2^ in the tibial and mandibular periosteum [13,51,52]. The mean value found in the calvarial periosteum was 120–160 cm/cm^2^, which is comparable to the values found in the control group in our study [18,30]. However, in the groups treated with alendronate, the functional capillary densities were significantly reduced; in the two weeks treatment group, it was approximately half and in the six weeks treatment group, it reduced to a third of the value in comparison to the control group. Arora et al. examined iris blood supply in bisphosphonate-treated rats and mice after systemic and local injection of lipopolysaccharides, setting free the inflammatory cytokines IL-1β and TNF-alpha accumulated in bisphosphonate-treated tissues [17,42,43,[53], [54], [55]]. They found that the iridial functional capillary density significantly decreased (and the dysfunctional capillary density increased) compared to controls as a reaction to the provoked inflammation. Differences in the exact reciprocal values of functional and dysfunctional capillary densities are considered to be caused by the opening of arteriovenous shunts, which are closed under physiological conditions [17]. Moreover, various other factors influencing microperfusion are under investigation, including an altered endothelium-dependent vasomotoric function induced by FPPS-inhibitors [[56], [57], [58]].

The results mentioned above corroborate the findings of our study, demonstrating a reduced functional capillary density in connection with an increased inflammatory response in the alendronate-treated groups. Moreover, intravital microscopy studies examining neoangiogenesis have shown the key role of vascular endothelial growth factor (VEGF) regarding the maintenance of vascularisation [59]. Bisphosphonates are known to inhibit VEGF [8]. Therefore, the reduced functional capillary density in the periosteum of the alendronate-treated animals in our study can be reasonably explained.

In accordance with reduced functional capillary density, periosteal healing was significantly impaired by alendronate in the present study. Periosteal healing is a complex process requiring adequate blood supply, neoangiogenesis and the presence of several factors such as FGF and VEGF. Bisphosphonates are known to inhibit angiogenesis by interfering with endothelial cell proliferation, limiting circulating endothelial progenitor cells (CEPC), reducing VEGF production and increasing endothelial cell apoptosis [8,44,60,61]. We observed several of these conditions for impaired healing due to reduced functional capillary density and increased inflammation. This is in accordance with the results reported by Zhang et al. and Schaser et al. who examined the healing of standardised closed fractures and severe closed soft tissue injuries in the tibia of rats. Both studies showed long-lasting impairment of periosteal microcirculation and inflammatory reaction at the site of trauma [62,63]. Taken together, our findings of increased inflammation and decreased functional capillary density in the periosteum under alendronate treatment, and the characteristics of bisphosphonates mentioned above, suggest that impaired periosteal defect healing is absolutely comprehensible.

The present study has some strengths and limitations. The primary strength of the study is the in vivo detection of reduced periosteal perfusion and delayed periosteal healing under alendronate therapy. According to these findings, a possible pathomechanism of MRONJ could be identified, a disease with a rapidly increasing number of cases. Second, we could present a model and study design for simultaneously investigating several in vivo parameters, such as periosteal microhemodynamics, inflammation and wound healing within the same experimental series, thereby reducing the number of animal experiments. Moreover, the method enables further studies to compare the effects of other bisphosphonates, antiresorptive and antiangiogenic drugs.

A limitation of the study could be that in contrast to the long-term bisphosphonate therapy for osteoporosis and cancer patients, the period of alendronate therapy in the animal experiment presented here was comparatively short. Alterations in periosteal perfusion and defect healing may have been even more remarkable and conclusive with longer periods of alendronate administration. In addition, the relatively small number of experimental animals used could be considered a limiting factor. Nevertheless, we detected significant differences in the essential parameters of periosteal perfusion and periosteal defect healing. A technical limitation is that venules could not be measured in an identical plane using microscopy at any point of investigation, which led to small differences in the examined vessel diameters. Finally, the periosteum of the calvaria was investigated, instead of the jaw. However, the setting of a jawbone chamber, if at all possible, would have required a study design with larger animals, such as sheep, which is not justified in our opinion with regard to the available data.

In conclusion, we demonstrated a significant negative impact of the bisphosphonate alendronate on periosteal microperfusion in vivo with respect to inflammation, functional capillary density and defect healing. Subsequent studies using other bisphosphonates or antiresorptive drugs may confirm these results. With regard to the crucial role of an efficiently perfused periosteum in healthy bone metabolism, future research should focus on the impairment of periosteal microperfusion under bisphosphonate treatment to reduce the risk of MRONJ.

## Author contribution statement

Danielle Nicole Kundert: Analyzed and interpreted the data; Wrote the paper. Frank Tavassol, Prof. Dr. Dr.: Conceived and designed the experiments; Analyzed and interpreted the data. Andreas Kampmann, Dr.; Nils-Claudius Gellrich, Prof. Dr. Dr.: Conceived and designed the experiments; Analyzed and interpreted the data; Contributed reagents, materials, analysis tools or data. Daniel Lindhorst, Dr. Dr.; Paul Schumann, PD Dr. Dr: Conceived and designed the experiments; Performed the experiments; Analyzed and interpreted the data. Marc Michael Precht: Analyzed and interpreted the data.

## Data availability statement

Data included in article/supp. Material/referenced in article.

## Declaration of competing interest

The authors declare that they have no known competing financial interests or personal relationships that could have appeared to influence the work reported in this paper.
